# Next stop – mental health: a qualitative study of healthcare journeys from the perspective of young adults in Sweden

**DOI:** 10.1186/s12913-025-12510-5

**Published:** 2025-03-12

**Authors:** Katrin Häggström Westberg, Katerina Cerna, Mikael G. Ahlborg, Julia S. Malmborg, Petra Svedberg, Lena Petersson

**Affiliations:** 1 School of Health and Welfare, Halmstad University, Box 823, Halmstad, SE - 301 18 Sweden; 2 School of Information Technology, Halmstad University, Box 823, Halmstad, SE-301 18 Sweden

**Keywords:** Young adults, Help-seeking, Mental health, Metaphors, Healthcare journeys, Agency, Qualitative

## Abstract

**Background:**

Help-seeking for mental health problems is a complex process that involves handling both personal challenges and dealing with the organizational structure of the healthcare system. The healthcare system is siloed and fragmented, but it is unclear how the challenges are experienced by the young adults and what their healthcare journeys look like. Therefore, the aim of this study was to explore experiences of young adults’ healthcare journeys in the context of help-seeking for common mental health problems.

**Methods:**

In total, 25 young adults (16 women and 9 men) from a student healthcare centre at a Swedish university seeking help for common mental health problems, such as anxiety and depression, were interviewed. A qualitative thematic analysis with an inductive approach was done, and results were abstracted and presented in terms of journey-related metaphors.

**Results:**

The healthcare journeys of young adults were described as Taxi Riding, Commuting, Sightseeing, and Backpacking. *Taxi riding* and *Commuting* are defined by going in a straightforward and smooth way in the healthcare system, without major obstacles to care. In contrast, *Sightseeing* and *Backpacking* are characterized by more diffuse and negative experiences, where the young adults are not satisfied with the help received from healthcare providers. Help-seeking is not conformant with the design of the healthcare system but steered by a range of factors, including individual experiences and young adults’ agency, the available resources at the various healthcare providers, and interaction with healthcare professionals.

**Conclusions:**

Young adults’ healthcare journeys in the context of help-seeking for common mental health problems are related to individual, relational, and organizational factors. Some journeys run smoothly, epitomizing a functioning healthcare system that accommodates a rational help-seeker. Other journeys depict a rigid healthcare system, where the success and nature of the journey primarily depend on individual agency and on not becoming discouraged by obstacles. There is a need for more knowledge on how to support young adults’ mental health help-seeking. However, we also need more insights into how the healthcare system can become more receptive and accommodating toward the needs of young adults with common mental health problems.

**Supplementary Information:**

The online version contains supplementary material available at 10.1186/s12913-025-12510-5.

## Background

Mental health problems often first appear during adolescent years and early adulthood [1]. Common mental health problems for young adults are depression, anxiety [2, 3], and substance use disorders [4]. Recurring episodes and long-term untreated mental health problems in adolescence lead to poorer prognosis further on in life, with increased risk of complex and persistent mental health problems as an adult [5]. Despite the supporting evidence for early interventions [6], young people with mental health problems worldwide struggle to access appropriate and timely care [7].

Previous research on the help-seeking process has largely focused on individual-level barriers, such as lack of knowledge about where to seek professional help [8, 9], stigmatization [8–12], or preferences for self-management of problems [8, 10]. The young adult is required to be a rational and competent actor when encountering mental health problems, correctly judging the nature and severity of mental health problems and accurately seeking help at the ‘right time’ and at the ‘right level’ of care. However, help-seeking is in fact a dynamic and psychosocial process dependent on multiple factors and not only a point-to-point transition [13]. For example, seeking professional help usually involves numerous points of contact and prolonged waiting times [14, 15]. The healthcare system, in various contexts, is experienced as inaccessible, unresponsive, and inflexible [13]. This complexity warrants viewing the help-seeking process for mental health problems as a healthcare journey, rather than a point-to-point transition by a rational actor.

The difficulties providing timely care and access for mental health problems seem pervasive internationally [16]. In the Swedish healthcare system, young adults with mental health problems can turn to a variety of healthcare settings, such as primary care units, youth guidance centres, student healthcare services, and specialized care, such as psychiatric units [17]. Primary care is regarded as a “first line care”, with a particular task of providing person-centred and integrated care (God och Nära vård), this also includes mental healthcare [18]. In young adulthood, mental health problems are often characterized by a fluid and mixed symptomatology. However, care and treatment for common mental health problems is traditionally organized according to single-disorder observations and diagnostic thresholds. This organization of ‘siloed care’ fails to account for multifactorial mental health problems, commonly present in young adulthood [19]. Thus, a siloed and fragmented healthcare system poses challenges for young adults in need of healthcare for mental health problems. However, it is unclear how the challenges are manifested and experienced by the young adults and what their healthcare journeys look like. The voices of young adults, particularly on the issue of how organizational factors of the healthcare system affect the help-seeking process, are limited, but necessary in order to gain insight into reasons for lack of support and care [13].

Therefore, the aim of this study was to explore the experiences of young adults’ healthcare journeys in the context of help-seeking for common mental health problems.

## Methods

### Study design

We employed a qualitative inductive design, combining thematic analysis [20, 21] and metaphorical analysis [22], to explore the healthcare journeys of young adults, between the age of 18–30, seeking help for common mental health problems such as anxiety and depression. This approach allowed us to systematically and inductively identify and interpret key themes that emerged from participants’ experiences. Describing the experiences of the healthcare journeys through metaphors provided deeper insights into the complex and often nuanced ways in which young adults conceptualize and navigate mental health care. We aimed to capture both the experiences and the underlying, symbolic meanings within participants’ help-seeking behaviours. To ensure trustworthiness, the study is reported in accordance with the Consolidated Criteria for Reporting Qualitative Research 32-item checklist [23].

### Setting

The study was conducted at a university in a county council area in the south of Sweden. The Swedish healthcare system is publicly financed based on local taxation; residents are insured by the state. Healthcare responsibility is decentralized to 21 county councils, whose responsibilities include healthcare provision and the promotion of good health for citizens. In addition, individuals who are students at a Swedish university can also contact the student healthcare centre at the university. The student healthcare centre offers free-of-charge counselling sessions to students regarding their health and well-being. They also help students by referring them to publicly available healthcare in Sweden in non-study-related healthcare matters.

### Participants

Young adults between ages 18 and 30 seeking help with mental health problems from a student healthcare centre were invited to participate in the study. The young adults had in many cases sought help from several healthcare providers in the healthcare system. Healthcare personnel from the centre aided in the recruitment phase, by informing young adults seeking help for mental health problems about the study. The young adults received brief written information about the study and an envelope. They indicated their interest in participating in the study by placing the paper with their desire to participate in the envelope, sealing it and handing it in to the personnel. The sealed envelopes were then forwarded by the healthcare personnel to one author (LP). This ensured that the healthcare personnel remained unaware whether the students accepted or declined participation in the study. We received 34 envelopes from students that all expressed an interest in participating. After contacting all 34, in total, 25 young adults were eventually interviewed; 16 women and nine men, aged 20 to 30.

### Data collection

The individual, semi-structured interviews were performed either at the university campus or by video call (using either the Teams or Zoom platforms), between December 2021 and January 2023 by LP (PhD and senior lecturer in pedagogy with previous experience of interviewing) and SL (PhD and senior lecturer in informatics with previous experience of interviewing). The researchers had no prior professional relationship with the young adults, such as serving as teachers. The interview started with the introductory question, “Can you tell me about your experiences of seeking care for mental health problems?” Follow-up questions included where and when they had sought help, what professionals they met when seeking help, and their experiences of the healthcare or support given, see Interview guide developed for this study (Supplementary file A). Each interview ranged between 19 and 67 min, and the total time was 17 h and 2 min, with a mean of 41 min per interview. The interviews were audio-recorded and transcribed verbatim.

### Data analysis

We used an inductive approach to analyse the empirical data, consisting of three main steps: (1) thematic analysis of interviews, (2) development of metaphors for healthcare journeys, and (3) development of themes, illustrated in a model depicting differences between the four healthcare journeys. The themes are related to young adults’ experiences and agency, interaction with healthcare professionals and interaction with healthcare system.

In step 1, a thematic analysis was performed to explore university students’ experiences of healthcare journeys in relation to help-seeking for mental health problems. The basis for choosing thematic analysis was a desire to unravel the complexity of the socially constructed process of mental health help-seeking in an inductive way. The analysis followed the steps of analysis as described by Braun and Clarke [20, 21]: (1) Data familiarization and writing familiarization notes; (2) systematic data coding; (3) generating initial themes from coded and collated data; (4) developing and reviewing themes; and (5) refining, defining and naming themes. First, each author read through the transcribed interviews separately, and then all met during short sessions, to discuss the initial impression of the dataset. This resulted in familiarization, with the use of notes and mind-maps. Second, the systematic data coding was conducted, by KC, KHW, MA, JSM, and LP in ATLAS.ti web v5.4.0 (Scientific Software Development GmbH, Berlin, Germany). No AI-supported automated coding was performed in the software. The initial coding was done separately by the research team members, who then jointly met to ascertain alignment in the research process. During this phase of inductive coding, it was noted that codes regarding help-seeking ranged from descriptions of individual problems and barriers pertaining to the healthcare organization, to both lack and use of various resources and positive experiences. However, it was apparent that help-seeking was described as a transitional process by young adults. This insight led to the use of tentative and initial themes pertaining to help-seeking travels, individual agency and interaction with healthcare professionals and the healthcare system.

In step 2, we developed, defined, and named the themes during an intensive analysis period, with recurring meetings amongst the research team. The research team delved deeper into the preliminary findings, to move beyond surface-level insights toward a synthesis of abstract representations. Both semantic and latent content were used in the analysis [21]. During this intense analysis work, the use of travel metaphors seemed useful to elucidate the essence of the transitional process described by the young adults. Various metaphorical forms were discussed, and the abstract metaphors that were chosen finally resulted in concrete descriptions of four different typology journeys, each of which has distinctly different features [22]. Quotations from the interviews were chosen to illustrate and add depth to the descriptive text in each theme/metaphor.

Finally, in step 3, we analysed the four healthcare journeys, matching the characteristics of each journey to three themes relating to the help-seeking process. The purpose was to understand and describe how the experiences and interactions of young adults in the four journeys differ from each other in relation to the three themes *Individual Experience and Agency*, *Interaction with Healthcare Professionals*, and *Interaction with Healthcare System*. This final part of the analysis process was conducted initially by two authors (KHW and JSM), and PS and LP thereafter developed the model further. Two authors (MA and KC) acted as co-assessors, and the results were discussed continuously with all authors until a consensus was reached. Finally, all authors reviewed and discussed the analysis, to increase trustworthiness and rigor. To further strengthen trustworthiness, the young adults’ quotations used in this paper were translated from Swedish to English by a native English-speaking professional translator and were edited only slightly to improve readability.

## Results

The findings, based on young adults’ experiences, are presented as four distinct metaphors that emerged from the analysis, each illustrating a different healthcare journey. The journeys do not represent individual interviews but should instead be understood as typologies reflecting the most prominent aspects of the participants’ narratives, with key differences emphasized to distinguish each metaphor. Many young adults may feel familiar with features of more than one of the journey metaphors, however, they might recognize their experiences most strongly in one of them. This approach was purposefully chosen, to highlight the complex realities that young adults encounter when navigating the Swedish healthcare system. At the end of this section, a model is presented that illustrates key characteristics of these healthcare journeys, focusing on the participants’ experiences and agency, interaction with healthcare professionals, and interaction with the healthcare system.

### Taxi riding

This metaphor is used to represent a journey where young adults contact a healthcare unit that fulfils their needs and takes them successfully from point A to point B. It represents an objectively ideal journey, with few obstacles and easy access to care. They knew where to initiate contact and were met by healthcare professionals who recognized their needs and could provide them with sufficient help. The journey is characterized by satisfaction and typically includes one or a few episodic sessions.

After realizing they need care, the journey is steered by young adult’s ability to choose their starting point. Although the starting point may vary, depending on what type of mental health problems are experienced, they know where to turn to get help swiftly, and can navigate the system. The taxi riding journey is defined by its immediacy and smoothness:

*Getting care is easy. When I sought help*,* I got an appointment immediately and it was great*,* it helped me a lot. //She encouraged me to start forgetting about things and really focusing on taking the initiative to meet friends*,* getting in touch with people. I did that straight after my session with her and I felt that it helped a lot. IP7*.

This quotation shows that young adults felt that their needs were recognized and that they were provided with strategies that they could immediately adopt in their everyday lives. Young adults might have met the healthcare provider only a few times, so the most important step was the establishment of the first contact. In some cases, the healthcare provider saw a need to refer young adults to another provider. This was done with the reassurance that young adults had a possibility to return to the first contact, in case the next health provider was unable to fulfil their needs.

*They said that my stress and anxiety had more to do with my studies. And they told me “If you don’t get help from student healthcare*,* get back in touch with us”. IP12*.

The smoothness of access and acquired knowledge during the journey leads to a feeling of security and comfort to continue their journey, if they should choose to do so.

Taxi riding captures the journey at a moment when the help-seeking adults perceive the help as satisfactory, leading to the desired outcome of feeling better. However, for some of those interviewed, the smoothness was rather linked to some steps they chose not to take, like choosing not to initiate contact after being referred to a different provider but rather utilizing one’s own resources. When asked about other forms of contact with healthcare (other than the Student Healthcare Centre), one young adult said:

*…I tried. It’s really hard to get an appointment*,* it takes a very long time*,* and first you have to meet with some healthcare person and then you… So I thought*,* well*,* I’m doing my bit now. So I’ve started listening to audio books a lot and I have started googling*,* I ‘ve started doing my own things to get out of there*,* because I’ve already been… I know what the process looks like. So no*,* unfortunately I haven’t [sought help]*,* because it’s going to take a really long time and I don’t know how I’ll feel then. IP7*.

The taxi riding journey is a characterized by needing help once and receiving it effortlessly. This type of journey is defined by the fact that young adults experience no or little trouble getting the care they need. The taxi ride is also the “most ideal”– the patient gets into the taxi, reaches the destination/healthcare organisation, gets help, and leaves.

### Commuting

The commuting journey is characterized by young adults experiencing a mental health problem and having sporadic or episodic contact with healthcare for a prolonged period of time. The contact is often initiated rather effortlessly and constitutes an overall positive experience. Young adults can formulate their problems in a way that facilitates the assessment of their needs. This is made possible by shared knowledge, often gained from family or friends with experience of mental health problems, alternatively working in the healthcare sector which helps guide young adults to the right level of care. The first contact is often initiated after young adults confide in a family member or someone close to them who encourages them to seek help. The journey builds on continual contact between young adults and their provider.

As young adults become aware that their symptoms re-emerge on a continual basis, there is a realization of a need for regular contact. Early on, it becomes obvious that continual contact is suitable, and with that, a relationship starts to form.

*No*,* but I think it’s been relatively fast. I don’t remember*,* but what could it have been? Hardly a week after I called the Primary care unit until I got my first appointment. And then we scheduled talks successively*,* with one or two weeks in between. And sometimes it was in person and sometimes phone calls. And at [clinic name] the same thing*,* sometimes it was two weeks in between and sometimes perhaps four*,* but it was still regular. And XXX (online health-care provider) as well*,* we booked appointments that suited us. IP11*.

Commuting begins as young adults perceive the care to be adequate and become comfortable with the provider, while simultaneously gaining an understanding of the healthcare system. The help-seeking and contact with care is experienced as effortless. Commuting contains episodic but recurring contact between the help-seeking adults and the healthcare professionals. This recurring contact is often interpreted as being positive for young adults with mental health problems. Knowing that a provider is available whenever they have a need increases young adults’ sense of comfort.

*“[Healthcare professional] said to me that “if you feel bad*,* come over and I’m sure we can talk for a bit… have a ten minute talk or a quick coffee together or I’ll have a short break or something like that”. And the thing that she said it helped*,* then I knew that ok*,* I’m not alone. IP3*.

The commuting journey does not need to be confined to one destination but follows the healthcare system’s organization and the various needs of young adults as they develop and age. A sense of security or comfort forms due to familiarization with the healthcare system and built-up trust with the care provider transfers between different care settings.

*Because*,* at the clinic for eating disorders*,* they talk to people who have eating disorders the whole time. So they had seen this before*,* there was nothing that was new to them. They had a really good understanding and the same thing at Child- and Adolescent Psychiatry. I got the feeling that they were specialized in exactly what I was talking about. IP1*.

In this journey, referral to another care provider, or having multiple simultaneous contacts, is perceived as unproblematic and straightforward. As young adults gain experience in the commuting journey, they gain a better understanding of their own of symptoms and acquired strategies for self-management.

The commuting journey starts when young adults contact a care provider at the appropriate level of care according to how the healthcare system is organized, and the healthcare professionals at these units understand their need for continual treatment. If young adults understand the structure of the healthcare system, commuting back and forth, sometimes between different care providers is facilitated. Over time, the “commuter” can develop into an autonomous and self-confident care recipient with tools to manage, in part, their mental health problems between visits, but also an ability to recognize more severe symptoms.

### Sightseeing

This metaphor is used to represent a journey that resembles a passive tourist going to different destinations with little influence over what sights to see, how long each stop will be, and whether access is granted at all stops. From the perspective of young adults, this journey is poorly organized, difficult to understand, and exhausting from waiting in line, not feeling welcomed by the healthcare system, and sometimes being denied access, which can result in a sense of resignation - “no one can help me.” The first contact with healthcare providers, whether it is at primary care or elsewhere, is often a negative experience, characterized by rejection by the care provider or immediate referral to another provider, leading to a sense of confusion.

*“I got the answer from the healthcare professional: “You need help that we can’t offer*,* and we don’t want to dig into things we cannot finish.” So*,* then*,* they sent a referral to a psychiatric unit and the psychiatric unit said “we can’t take her”. They sent me back to the Youth clinic. Then the Youth clinic sent me to Primary care and Primary care said the same thing; “we can’t take her*,* we can’t offer her any help. We don’t have the resources or the knowledge”. Then they sent me back to the Youth clinic and the Youth clinic eventually referred me to the psychiatric unit once again. So I ended up going there twice. Not to a psychologist. The first time was to a nurse and then to a doctor. And here I am*,* three months later*,* and I have no contact with the psychiatric unit*,* no contact with the Youth clinic and only recently after several ifs and buts got an appointment with a counsellor at the primary care clinic. IP15*.

Young adults were sent around from one service to another and back again, without receiving access to care. In some cases this was related to miscommunication whilst young adults had difficulties describing their mental health problems. A lot of time was spent waiting for responses from various healthcare settings. Young adults experienced that transitions between different providers entailed miscommunication and lost referrals, which sometimes forced them to take action to make sure their case had been received. If the referral was lost, they must contact their previous care provider to see where a mistake has been made.

*She was supposed to send a referral before she started to work elsewhere*,* but she never did. Or something happened*,* so it never got there… When I called Psychiatry*,* they hadn’t received anything. IP6*.

Long waiting times between appointments were seen as problematic when mood and mental health problems fluctuate. Young adults expressed a need for help now rather than later because they knew how they felt but were uncertain how they would feel in weeks or months. Being sent around made young adults begin to wonder if they were even on the “right tour”. Self-doubt and feelings of hopelessness developed, both due to a lack of validation by individual healthcare professionals and the rejection by different entities within the healthcare system. Young adults started to wonder whether the problem was within themselves or because of a lack of personal chemistry with the healthcare professionals they meet. Being sent around also meant that young adults had to tell their story repeatedly, which was a wearisome process and by some, described as the worst part of the journey, especially if they had attempted suicide when they were younger.

*I start over every time. Now I’m about to meet a new counsellor and get praise again. I’ve done this process for several years. I never get further than the first step. And it sucks. The hardest thing is to start telling the whole story from the start again. IP15*.

Another part of the journey described by young adults was the initiation of a care contact that seemed promising at first, but for some reason, this contact was terminated. Named reasons were restrictions, such as reaching the maximum number of appointments allowed, or age limits of certain healthcare providers. Other reasons for rejection and termination of care contacts were described as an unwillingness or inability of the healthcare professional to provide the care that is correct, according to the young adults. Young adults articulated disbelief and scepticism toward the healthcare system because of the perceived lack of help. The consequence of the sightseeing journey is that young adults only start to scratch the surface of their mental health problems and their road to recovery. The sightseeing tour is characterized by young adults experiencing a lack of control, confusion about the healthcare system, and a sense of resignation.

### Backpacking

The backpacking metaphor represents individuals who toil through the healthcare system, mainly driven by their own agency and needs, yet unable to find a place to settle down and get help. The journey is shaped by the discrepancy between young adults’ needs and the structure and resources of the healthcare system but is also highly defined by young adults’ agency and personal choices within – and adaptation to – the system. The backpacking journey involves a mix of episodic and continuous contacts and self-initiated breaks, taken independently of recommendations and prescriptions from healthcare providers.

*Many times*,* I have had to break the contact myself because I have felt that it has not given me anything*,* nor that the people I have met have really understood. IP24*.

The backpacking journey is typically initiated at a young age, starting off with a negative experience of seeking care for mental health problems. Young adults are either dismissed because the provider determines that they are not ill enough, or because their concerns are not taken seriously.

*And then I had to fill in a form about depression and after that the first thing they said was “yeah*,* we want you to start on medication” and I didn’t want that*,* and then they literally said “well*,* in that case we can’t help you” and that didn’t feel good. IP18*.

The backpacking journey does not go in one linear or straightforward way, but splits into multiple pathways, because young adults seek help from multiple healthcare providers, often at the same time.

*And now I have been calling back and forth and here and there and tried to find a psychologist and been in contact with private psychologists and I have been in touch with my primary care clinic. IP10*.

Young adults reported unmet needs such as not being offered conversational therapy when they had an ongoing medication, leading them to act. These kinds of additional contacts were not easily accessible and required efforts by young adults calling several caregivers or sending them messages or emails. Young adults on a backpacking journey expressed impatience and frustration about long waiting times and standardized care regimes and experienced not being regarded as someone with individual needs. In the backpacking journey, young adults’ dissatisfaction with inefficient care drove them to keep searching, rather than accepting what was offered. After a period of continuous struggles with their mental health, young adults sometimes decided to go back to a previous healthcare provider or used self-referrals to other healthcare providers.

*I felt so bad that I contacted healthcare again after a few months and then I contacted primary care. It wasn’t so good there*,* because there they had 25-minute meetings. I was only given a note to take home*,* and I said*,* “But I need help with a referral to adult psychiatry” and I gave her all my electronic health records. She just said “No*,* you don’t need that.” Then I tried to refer myself… but I only got the answer that you must go through your primary care centre”. IP2*.

In the backpacking journey, the traveller is resourceful and has a high level of agency, exploring all possible avenues within a predefined healthcare system. However, for many young adults, the journey seldom led to satisfactory results. They frequently found that the competence and resources of healthcare providers did not match their needs, and that young adults often themselves needed to recognize that the provider was not the right for them. Unsuccessful encounters with healthcare providers required effort, which placing an additional burden on young adults, already dealing with their mental health problems.

### A model that summarizes the characteristics of the healthcare journeys

**Fig. 1:**
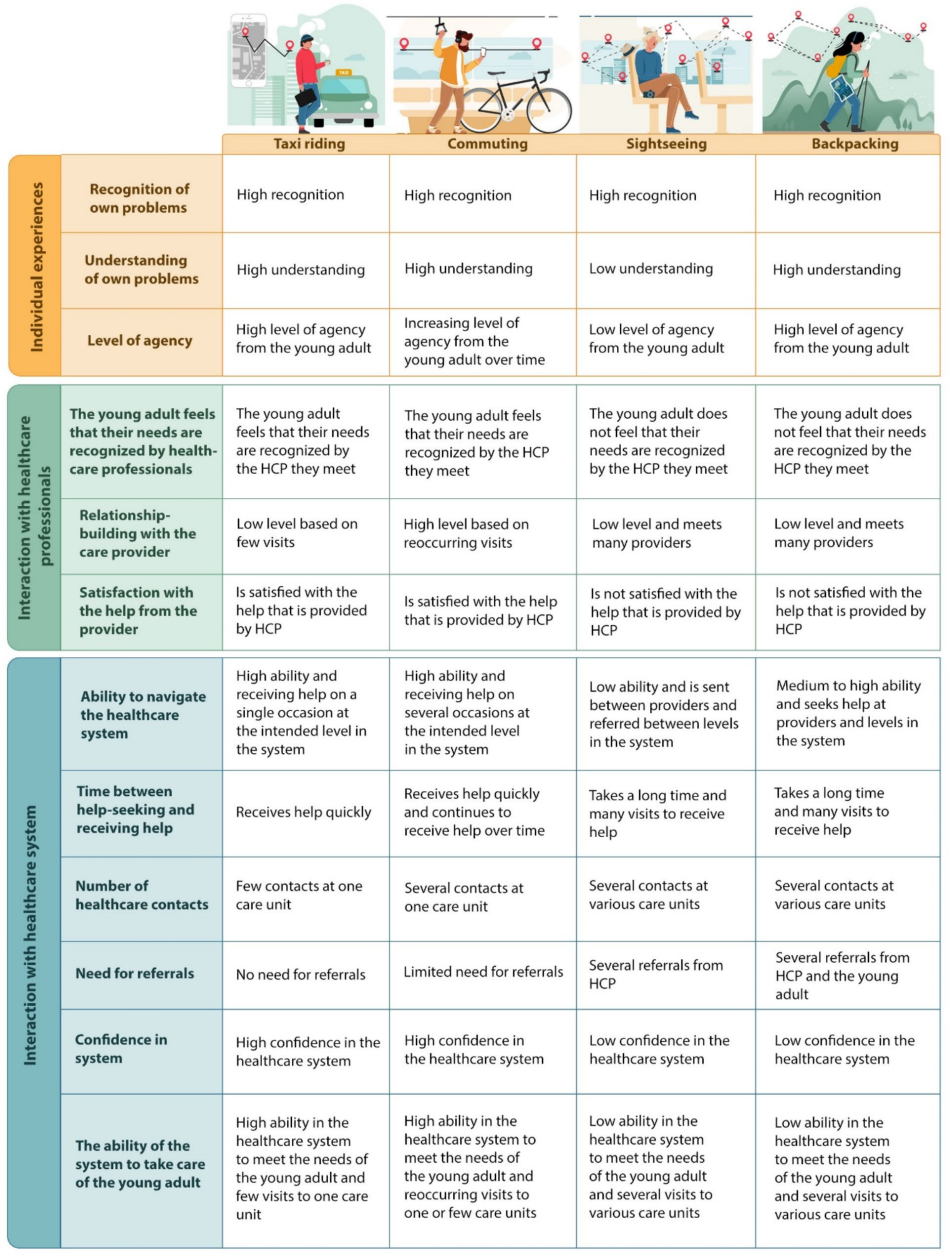
Summary of the four healthcare journeys’ characteristics. HCP, healthcare professionals

Based on the findings in steps 1 and 2 of the analysis, we propose a model to summarize the characteristics of the healthcare journeys (Fig. 1). This model summarizes the young adults’ perspectives in relation to the themes *Individual Experience and Agency* (row 1–3), *Interaction with Healthcare Professionals* (row 4–6), and *Interaction with the Healthcare System* (row 7–12). We can see in the findings that experiences of young adults’ healthcare journeys in the context of help-seeking for common mental health problems are related to, respectively, individual, relational, and organizational factors.

The model in the figure highlights the differences and commonalities among the different journeys: *Taxi riding* and *Commuting* are defined by going in a straightforward and smooth way in the healthcare system that works for both young adults and healthcare professionals. In contrast, *Sightseeing* and *Backpacking* are characterized by much more diffuse and negative experiences, where young adults are not satisfied with the help from the healthcare providers, and the results indicate that there is a mismatch between the resources in the healthcare system and young adults’ help-seeking for common mental health problems.

## Discussion

The principal findings showed that young adults’ experiences in seeking mental health support vary, as illustrated by four travel-related metaphors. These metaphors highlight the varying experiences and outcomes of their healthcare journeys.

*Taxi riding* represents an ideal help-seeking journey. Young adults understand where to turn and how to obtain the necessary help. This journey is smooth and efficient, characterized by quick access to a provider and care that is meaningful to young adults. This type of journey often results in high satisfaction, as the care provided matches their needs and expectations well. The journey is typically marked by high satisfaction as it unfolds in a straightforward manner with minimal obstacles. *Commuting* also offers a smooth experience but differs from taxi riding in its temporal aspect. It involves recurring, continuous contact with healthcare providers over a longer period. In contrast to *Taxi riding* and *Commuting*, both *Sightseeing* and *Backpacking* are marked by complexity and inefficiency. In the *Sightseeing* journey, young adults experience a “ping-pong” effect, being shuffled between various providers without achieving meaningful progress. This journey reflects a high degree of dissatisfaction, a lack of meaningful help and a low degree of individual agency. The fourth and final metaphor, *Backpacking*, is characterized by a strong sense of agency by young adults. They actively navigate and partially adapt to the healthcare system’s structure, seeking out multiple providers and persistently pursuing solutions. The backpacking journey is complex, taking young adults to several providers who do not automatically or always provide them with help. However, frustration with the situation serves as a further motivation that drives young adults to continue the journey.

Thus, our findings show that each healthcare journey is unique, reflecting different aspects of individual experience and agency, interactions with healthcare professionals, and interaction with the healthcare system. Each journey shows varying levels of personal agency, as well as distinct patterns of interaction with providers and the broader healthcare framework. The following discussion will highlight these journeys through the respective lenses of individual experience and agency, interactions with healthcare professionals, and interactions with the healthcare system.

### Individual experience and agency

Our findings relate to aspects of individual agency, competence and experience. The care that young adults are provided with is heavily dependent on their ability to demonstrate their mental state in a way that healthcare professionals can interpret. This entails that young adults also need to possess knowledge on the multidimensional concept of mental health, being able to make an accurate enough judgment on if, and when, to seek help, and they also need to know which healthcare provider to contact with the particular problem. Competence differentiates the four metaphorical journeys, with the sightseer apparently possessing the least capability. Other factors of importance in how the help-seeking journey develops are personal effort and individual experience. Personal effort constitutes a marked difference between journeys, where young adults on a backpacking journey relentlessly adapt to the fixed healthcare system and attempt to utilize it to their needs. By contrast, those on a sightseeing journey end up with scepticism and a sense of resignation. However, effort is related to the concept of resilience, which may be positively affected by mental health promotion interventions [24].

Individual experiences, and or support from others, were sometimes essential for the journeys. This is in alignment with the Network Episode Model of healthcare uptake and compliance. The Network Episode Model also raises the issue of agency, suggesting that individual agency, particularly as it relates to social processes, is an important aspect of healthcare pathways [25]. Our findings also suggest that previous experiences of mental health problems and help-seeking by others within their social networks, could contribute to a more smooth and efficient help-seeking journey. Another insight worth discussing is that young adults sometimes refuse the help that is offered. This occurrence might seem contradictory to their situation, given that they are seeking help. There are several possible explanations for this; it might be a case of unmet expectations, which can lead to young adults turning away from the care offered. Rejection of care might also be attributed to a lack of competence in young adults, i.e. an inability to take advantage of the care and resources offered. Another possible explanation is that refusal might be attributed to an expression of agency – young adults possess competence and know what will, and will not, work for them.

### Interaction with healthcare professionals

Relational factors impacting help-seeking journeys can largely be discussed within the realm of interaction with healthcare professionals. In the current study, young adults expressed that their individual treatment needs and preferences were not met in a satisfactory manner. The results may be discussed from the concept of person-centred care. In an integrative review by Pinho et al. [26], several indicators supporting person-centred care for patients with anxiety and depression are listed, such as individualized, flexible, and dynamic care planning, shared decision making, and adequately meeting the patient’s individual needs and preferences. Moreover, healthcare professionals should engage in informing and educating the patient, managing adherence to therapy, and formulating relapse prevention strategies. Young adults in the current study, particularly those experiencing the backpacking type of journey, occasionally expressed that the lack of meaningful connection in the relationship with their caregiver was one of the reasons for turning down care. Empathy, skilled communication, and shared power and decision making are factors within person-centred care that are highlighted as important for fulfilling one of the four constructs in the nursing framework for the person-centred care [27]. The importance of communication and relationship building was highlighted by our participants who experienced the healthcare journey of Commuting, which in several cases led to satisfaction with treatment. Young adults on the Backpacking journey described that care providers did not display competence, which can be related to the construct prerequisites in the nursing framework for person-centred care. It is important that the care provider possesses professional competence in terms of knowledge, skills and a commitment to fostering person-centred care [27]. In the Sightseeing journeys, it was described that young adults were sent around without receiving access to appropriate care. This can be related to the construct of the care environment in the nursing framework for person-centred care. In the current study, it was highlighted that the healthcare organization did not fully support young adults in their struggle to receive help for common mental health issues, particularly those on the Sightseeing journey. The characteristics of healthcare journeys and their degree of complexity seem to lead to shifting outcomes in terms of patient satisfaction, which is highlighted as an important aspect and the final construct in the nursing framework for person-centred care [27].

### Interaction with the healthcare system

The clinical implications of these findings are primarily related to organizational factors. First, the complex journeys presented in our findings, i.e. backpacking and sightseeing, expose flaws in the healthcare system regarding mental health care coordination. Lost referrals, prolonged waiting times and “siloed care” evidently contribute to patient frustration and hopelessness; however, they may also have more serious consequences, such as discontinued or lack of care. The siloed character of the current state of the Swedish healthcare system contributes to the seekers not “matching” the well predefined categories of the system. Hence the same person may hear they are both “too sick” or “too healthy” depending on the healthcare setting. In addition, many mental health problems are combined with comorbidities – which are not addressed at one place. This is demanding for the help-seekers, as they need to interact with many healthcare providers. Improved care coordination has been advocated for decades [28] without any substantial improvement. However, the recent digital transformation and increased attention to the benefits of information-driven care [29] can support the development toward a personalized healthcare with more effective coordination that uses existing data in the healthcare system [30]. Thus, digital transformation could give the healthcare system the opportunity to develop more accessible and personalized, proactive mental healthcare that is driven by various forms of data, which could be a way toward person-centred and integrated care (God och Nära vård) [18] for patients with common mental health problems.

Finally, and connected to care coordination, we believe that the pitfalls revealed in these healthcare journeys presented in this paper show potential for improvement in many aspects of healthcare for common mental health problems. Patient navigation services, remote monitoring and self-care guides, decision support systems and personalized treatment plans, along with tools to facilitate enhanced patient engagement, are just some of the promising areas for development [31, 32]. Thus, it has been suggested that care managers take on the role of the “spider-in-the-web”, to alleviate the burden of managing multiple healthcare contacts simultaneously [33]. In addition, artificial intelligence (AI) could enhance the accessibility, efficacy, and ethics of mental healthcare, albeit they are also accompanied by rising privacy and data security concerns [34]. Accordingly, psychiatric care is a discipline that has been slow to adopt AI [35]. However, the discipline is unique in medicine as it relies solely on patients’ self-reports of their thoughts, feelings, symptoms, and social interactions [36]. Therefore, clinicians who meet patients with mental health problems will benefit greatly from AI [37], because AI has a transformative potential, including applications such as early detection of mental health problems, personalized treatment plans, and AI-driven virtual therapists [34]. Further research should focus on how an AI-powered healthcare system, where humans and machines work together to compose complex and adaptive service systems that promote mental health, could be developed.

### Methodological considerations

There are methodological limitations that need to be considered. In qualitative research, credibility, dependability, confirmability, and transferability are used to describe trustworthiness [38]. The purposeful sample of young adults, both men and women, with various experiences of seeking help for mental health problems strengthens the credibility. Furthermore, the researcher’s familiarity with the methodology enabled in-depth interviews and was another strength of the study. Dependability was strengthened using an interview guide, ensuring that all young adults were asked the same questions.

When young adults were invited to participate, it was their choice whether to participate in the interview on-site or remotely. Thus, interviews were conducted remotely via Microsoft Teams or face-to-face, which might be seen as a limitation. However, remote interviews using videoconferencing services like Microsoft Teams can be beneficial and even preferred [39]. Confirmability was enhanced by including selected quotations from the data, to illustrate and provide depth to the descriptive text in each journey. A strength of our study is that we conducted workshops with young adults who participated in the interviews to confirm the analysis and metaphors used. A limitation of the study could be that we did not have access to the participants’ electronic health records. Thus, we do not know how healthcare professionals describe the participants’ mental health and their healthcare journeys. Another limitation could be that all young adults were recruited from the student support service at one university in Sweden, which excludes young adults with other levels of education or with other professions or means of making a living (or being cared for). However, it is a strength of the study that the participants had various backgrounds; some did not have Swedish as a first language, some had immigrated to Sweden or had parents with immigration backgrounds from other parts of the world. Future research should focus on recruiting young adults from other settings and other countries. Yet another limitation could be that we did not analyse men and women separately, in order to identify differences between men’s and women’s healthcare journeys. Further research should focus on analysing gender differences in help-seeking for common mental health problems. Finally, it was a challenge to categorize young adults and their experiences as types of journeys, as they may have moved between different types of journeys, but ultimately, we believe that it is a strength that the journeys contain experiences from several young adults. We believe metaphors are considered a suitable method of portraying and making descriptions, as it is the journeys and not the people in focus in this paper, and they are easy to relate to and bring light to the complexity of young adults’ help-seeking for common mental health problems.

## Conclusion

To conclude, the journeys have distinct characteristics, with taxi riding and commuting running smoothly, epitomizing a functioning healthcare system that accommodates a rational help-seeker. These journeys work out smoothly because young adults meet the right people at the right time, and their needs are met. Sightseeing and backpacking journeys depict a rigid healthcare system where the success and nature of the journey primarily depends on individual agency and not becoming discouraged by obstacles. While help-seeking for mental health problems inherently happens at the individual level and between individuals, a functioning healthcare system should be readily available for all with mental health problems and not dependent on individual resources and level of agency. Thus, not only is further research on how to support young adults’ mental health help-seeking necessary, but also insights into how the healthcare system, with support from AI and information-driven care, can become more receptive and accommodate the needs of young adults.

## Supplementary Information

Supplementary Material 1.

## Data Availability

Empirical material generated and/or analysed during the current study is not publicly available but is available from the corresponding author on reasonable request.

## Abbreviations

AI: Artificial Intelligence
HCP: Healthcare professionals
