# Supplementary material for: Next stop – mental health: a qualitative study of healthcare journeys from the perspective of young adults in Sweden
Source: BMC Health Serv Res. 2025 Mar 12;25:364. doi: 10.1186/s12913-025-12510-5 (PMC11900026; doi:10.1186/s12913-025-12510-5)
Supplement: Supplementary file 1 — Supplementary Material 1. [file 12913_2025_12510_MOESM1_ESM.docx]

**Supplementary file A, Interview guide**

Describe your experiences of seeking help for mental health problems. Elaborate on who you met, where and when you sought help, and how you perceived the support and treatment.

Describe, according to your view, the structure of the healthcare organization and how it is matched to the needs of young adults with mental health problems.
